# Zika Virus, French Polynesia, South Pacific, 2013

**DOI:** 10.3201/eid2006.140138

**Published:** 2014-06

**Authors:** Van-Mai Cao-Lormeau, Claudine Roche, Anita Teissier, Emilie Robin, Anne-Laure Berry, Henri-Pierre Mallet, Amadou Alpha Sall, Didier Musso

**Affiliations:** Institut Louis Malardé, Papeete, Tahiti, French Polynesia (V.-M. Cao-Lormeau, C. Roche, A. Teissier, E. Robin, D. Musso);; Direction de la Santé (A.-L. Berry, H.-P. Mallet);; Institut Pasteur, Dakar, Senegal (A.A. Sall)

**Keywords:** Zika virus, outbreak, French Polynesia, South Pacific, viruses, flavivirus, vector-borne infections, mosquitoes

**To the Editor:** Isolated in 1947 from a rhesus monkey in Zika forest, Uganda, Zika virus (ZIKV) is a mosquito-borne flavivirus (*1*). For half a century, ZIKV was described only as causing sporadic human infections in Africa and Asia, which was mostly confirmed by serologic methods (*2*). In 2007, the first ZIKV outbreak reported outside Africa and Asia was retrospectively documented from biological samples of patients on Yap Island, Federated States of Micronesia, North Pacific, who had received an incorrect diagnosis of dengue virus (DENV) (*3*,*4*). We report here the early investigations that led to identification of ZIKV as the causative agent of an outbreak that started in October 2013 in French Polynesia.

French Polynesia is a French overseas territory located in the South Pacific. The ≈270,000 inhabitants live on 67 islands distributed into 5 archipelagoes (Society, Marquesas, Tuamotu, Gambier, and Austral Islands). Surveillance for acute febrile illnesses is coordinated by the Department of Health with the contribution of a sentinel network of public and private practitioners, the main public hospital (Centre Hospitalier du Taaone), and the public health and research institute (Institut Louis Malardé [ILM]). As part of this syndromic surveillance system, ILM has implemented protocols for detecting arboviruses that are known to cause outbreaks in French Polynesia, such as DENV, or that pose a risk for causing epidemics because of the presence of potential mosquito vectors. In addition, ILM provides DENV serotype identification for other Pacific island countries, including Yap State, as part of the regional surveillance of dengue (*5*). For that reason, a ZIKV reverse transcription PCR (RT-PCR) protocol by Lanciotti et al. (*3*) was implemented at ILM.

In October 2013 (week 41), a 53-year-old women (patient 1) and 2 other members of the household—her 52-year-old husband (patient 2) and her 42-year-old son-in-law (patient 3)—experienced a mild dengue-like illness consisting of low fever (<38°C), asthenia, wrist and fingers arthralgia, headache, and rash. Patients 2 and 3 also had conjunctivitis. Patient 1 had swollen ankles and aphthous ulcers. For all 3 patients, results were negative for DENV by RT-PCR and nonstructural protein 1 (NS1) antigen tests (*5*), for West-Nile virus by RT-PCR, and for chikungunya virus by RT-PCR; results of RT-PCR for ZIKV were equivocal for patients 1 and 2. During week 43, a 57-year-old patient (patient 4) reported similar symptoms; results of RT-PCR for DENV were negative, but results of RT-PCR for ZIKV were positive. ZIKV infection was then confirmed by sequencing of the genomic position 858–1138 encompassing the prM/E protein coding regions of ZIKV (GenBank accession no. KJ579441). The protocol was approved by the Ethics Committee of French Polynesia (reference no. 66/CEPF). Phylogenetic analysis of the sequence (Figure) showed ZIKV strain Cambodia 2010-FSS13025 (GenBank accession no. JN860885) as the closest strain (*6*).

**Figure:**
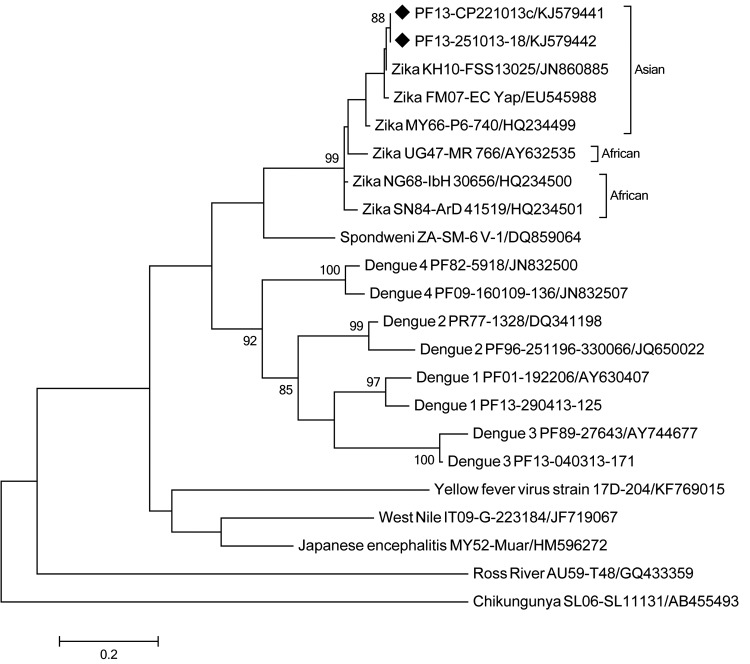
Phylogenetic analysis of partial M/E genes of 2 ZIKV strains, French Polynesia, 2013. The evolutionary history was inferred by using the maximum-likelihood method based on the Kimura 2-parameter model. The percentage of trees in which the associated taxa clustered is shown for values >85 next to the branches (1,000 replicates). Evolutionary analyses were conducted in MEGA5 (http://megasoftware.net/). Strains are labeled by country of origin and date-strain name/GenBank accession number. The 2 ZIKV strains collected in French Polynesia are marked with a black diamond. ZIKV, Zika virus. Scale bar indicates nucleotide substitutions per site.

Concomitant with these investigations, the Department of Health recorded an increased number of patients with a mild dengue-like syndrome and rash who were visiting primary care physicians. Given this information, we performed RT-PCR for ZIKV on 10 samples collected during weeks 43 and 44 from patients living in different archipelagoes that had tested negative for DENV NS1 antigen. Four samples gave negative results; 4, positive; and 2, equivocal. Sequencing of 1 ZIKV-positive sample from a patient in Nuku Hiva, Marquesas Islands (GenBank accession no. KJ579442) showed that it had 100% homology with the fragment sequenced from patient 4 who lived in Tahiti, Society Islands. The phylogenetic tree shows that the ZIKV that recently emerged in French Polynesia is similar to Cambodia 2010 and Yap State 2007 strains, which corroborates previous findings of an expansion of ZIKV Asian lineage (*7*,*8*). ZIKV was then isolated by inoculating Vero cells with RT-PCR samples positive for ZIKV. After 6 days of propagation, ZIKV-infected cells were detected by indirect immunofluorescence assay using specific hyperimmune mouse ascitic fluids provided by the Institut Pasteur (Dakar, Senegal) (*9*).

By week 51, the practitioners’ network recorded 5,895 patients with suspected ZIKV infections, leading to an estimate of 19,000 suspected cases when extrapolated to other care centers (adjusted to the mean consultation visits). Serum from 584 patients was tested by RT-PCR for ZIKV; 294 samples were positive.

This ZIKV outbreak is the largest documented and the first known to be caused by an arbovirus other than DENV in French Polynesia. To assess when ZIKV circulation in French Polynesia might have started, we will be conducting a retrospective study on DENV NS1 antigen–negative samples collected before the first ZIKV cases were detected. Investigations of the clinical features of ZIKV infections are ongoing. Particularly, because French Polynesia is experiencing concomitant ZIKV, DENV-1, and DENV-3 outbreaks, attention will be paid to whether sequential infections may affect disease outcome. Otherwise, because French Polynesia hosts several mosquito species, notably *Aedes aegypti*, already known to transmit ZIKV (*10*), but also other potential vectors, such as *Ae. polynesiensis*, human and entomologic surveillances have been reinforced to clarify the emergence factors of this outbreak.
